# Development and validation of a DWI-based intra- and peri-infarction radiomics model for predicting neurological deterioration in acute ischemic stroke

**DOI:** 10.3389/fneur.2026.1825520

**Published:** 2026-08-10

**Authors:** Pan Si, Ce Zong, Li Liu, DongQing Wang, Bo Song

**Affiliations:** 1 Department of Neurology, The First Affiliated Hospital of Zhengzhou University, Zhengzhou, Henan, China; 2 Department of Interventional Radiology, Zhengzhou Central Hospital Affiliated to Zhengzhou University, Zhengzhou, Henan, China; 3 Department of Radiology, Zhengzhou Central Hospital Affiliated to Zhengzhou University, Zhengzhou, Henan, China

**Keywords:** acute ischemic stroke, diffusion-weighted imaging, machine learning, neurological deterioration, peri-infarction zone, radiomics

## Abstract

**Introduction:**

Early prediction of neurological deterioration (ND) in acute ischemic stroke (AIS) remains challenging. We aimed to develop a diffusion-weighted imaging (DWI)-based radiomic model that integrates intra- and peri-infarction radiomic signatures with clinical characteristics to improve ND prediction.

**Methods:**

This retrospective study included 767 patients with anterior circulation AIS (training, *n* = 537; test, *n* = 230) who underwent DWI within 3 days of onset. Infarct cores were manually segmented, and peri-infarction regions (1–3 mm annular expansions) were generated. Radiomics features were extracted from all regions. Significant clinical predictors and radiomics features were selected to construct four models: clinical, intra-infarction radiomics, peri-infarction radiomics (1–3 mm), and a combined model. Performance was assessed using area under the curve (AUC), sensitivity, specificity, calibration, and decision curve analysis (DCA).

**Results:**

In total, 767 patients were included in the analysis. In multivariate analysis, glucose level was an independent clinical predictor of ND [odds ratio (OR) = 1.003; 95% confidence interval (95% CI), 1.001–1.006; *p* < 0.05]. Overall, 1,561 radiomic features were extracted from the intra-infarction region. Ten radiomics features were deemed valuable for dimensionality reduction and selection. Similarly, 1,561 radiomic features were extracted from the Peri2mm region, and 16 radiomics features were included. Among the intra-infarction radiomics models, the XGBoost model showed the best predictive efficiency, with AUCs of 0.911 and 0.898 for the training and test cohorts, respectively. The 2 mm peri-infarction model performed best among all peri-infarction models (AUCs: 0.854 for training, 0.850 for test). The combined model (intra-infarction + 2 mm peri-infarction + clinical feature) achieved the highest AUC values of 0.922 (training) and 0.911 (test), accompanied by favorable calibration and stable clinical net benefit. Although the combined model yielded a perfect sensitivity of 1.000 in the test cohort (95% Wilson CI: 0.867–1.000), this finding should be interpreted cautiously due to the small number of ND events (*n* = 25). Further pairwise comparison revealed that the combined model possessed significantly better discriminative ability than the clinical model and the 1 mm peri-infarction model in the test cohort (*p* < 0.05). However, the improvement over intra-infarction radiomics alone did not reach statistical significance in the test cohort (DeLong *p* = 0.179), and no significant superiority was observed versus the Peri2mm and Peri3mm models either.

**Discussion:**

Integrating DWI-derived intra-infarction radiomics, 2 mm peri-infarction signatures and clinical indicator achieves optimal AUC discrimination within the training cohort, though its statistical advantage over isolated intra-infarction radiomics could not be validated in the independent test set. This multimodal nomogram provides a non-invasive imaging tool to assist ND risk stratification for acute ischemic stroke patients, while larger multi-center cohorts are required to confirm its universal incremental predictive value and stable detection performance.

## 1 Introduction

Acute ischemic stroke (AIS) continues to be a major contributor to long-term disability and mortality globally, placing a significant strain on the healthcare infrastructure and socioeconomic productivity (1). A key challenge in the clinical management of AIS is the early identification of individuals at an elevated risk of neurological deterioration (ND)-a broad spectrum of neurological functional decline during hospitalization. Most prior studies focus on early deterioration within 24–72 h after stroke onset (2, 3), while our endpoint captures both early and delayed in-hospital neurological worsening. ND occurs in approximately 10–30% of patients with AIS (4) and is strongly associated with unfavorable functional outcomes, such as increased dependency on daily activities and higher in-hospital mortality rates (5). Therefore, accurate prediction of ND is clinically essential as it may facilitate timely interventions, including optimized hemodynamic management, tailored antiplatelet regimens, and intensified neurological monitoring, which can potentially attenuate further brain injury and improve long-term prognosis.

Diffusion-weighted imaging (DWI), a fundamental magnetic resonance imaging (MRI) sequence for diagnosing AIS (6), provides exceptional sensitivity in identifying the acute infarct core within minutes of ischemic onset (7). By measuring the restriction of water diffusion within ischemic tissue, DWI not only accurately localizes and delineates the infarct but also offers valuable indirect information regarding the underlying pathophysiology of tissue damage, such as cytotoxic edema and loss of cellular membrane integrity (8). The conventional clinical evaluation using DWI primarily involves qualitative appraisal, including infarct location and visually estimated size (9). However, these conventional approaches have a limited ability to detect subtle, spatially heterogeneous microstructural alterations that may precede the clinical manifestations of ND (10, 11). This underscores the need for more advanced data-driven methodologies to uncover prognostically relevant information embedded in DWI datasets.

Radiomics has emerged as a powerful computational approach in medical image analysis, allowing high-throughput extraction of numerous quantitative features, including spanning intensity, texture, and shape characteristics, from routinely acquired clinical images. These features can reveal intra-tissue heterogeneity at the subvisual level (12, 13). In the context of AIS, radiomic models derived from DWI have demonstrated the potential to predict various clinical endpoints, including functional recovery, hemorrhagic transformation, and infarct growth (14). Nevertheless, most existing radiomic studies on stroke have focused primarily on features extracted exclusively from the infarct core (intra-infarction radiomics) (15). Such a restricted scope fails to account for the peri-infarction zone, the metabolically unstable tissue surrounding the core infarct, which plays a critical role in the propagation of injury mechanisms, such as inflammatory responses, vasogenic edema, and blood–brain barrier dysfunction, all of which are implicated in the pathogenesis of ND (16).

Growing evidence indicates that the peri-infarction region contains biologically and prognostically relevant information that complements findings from the infarct core. In oncology, analogous strategies that integrate intratumoral and peritumoral radiomic features have substantially enhanced the predictive accuracy of models for treatment response and recurrence (11), highlighting the broader utility of radiomic analysis beyond the primary lesion boundary. Accumulating neuroimaging evidence has confirmed that the peri-infarction zone in acute ischemic stroke contains pathophysiologically distinct penumbral tissue with independent prognostic value. DWI-derived peri-infarct radiomic features can capture subtle microstructural alterations beyond the infarct core, providing incremental predictive power for neurological worsening and long-term functional outcomes (17). Despite these advances, the systematic incorporation of peri-infarction radiomics features into prediction models for ND in AIS remains underexplored. Moreover, no previous studies have employed a stepwise annular expansion approach to quantitatively assess spatial changes in the peri-infarction microenvironment.

To bridge this gap, the current study aimed to develop and validate radiomic models for predicting ND in patients with AIS using DWI data. In addition, this study aimed to build a combined radiomics-clinical model and evaluate its performance against that of a pure radiomics model. Such a tool could offer a non-invasive and clinically feasible means to support personalized treatment strategies and eventually mitigate the burden of neurological deterioration in AIS.

## 2 Materials and methods

### 2.1 Patient selection

**Figure 1:**
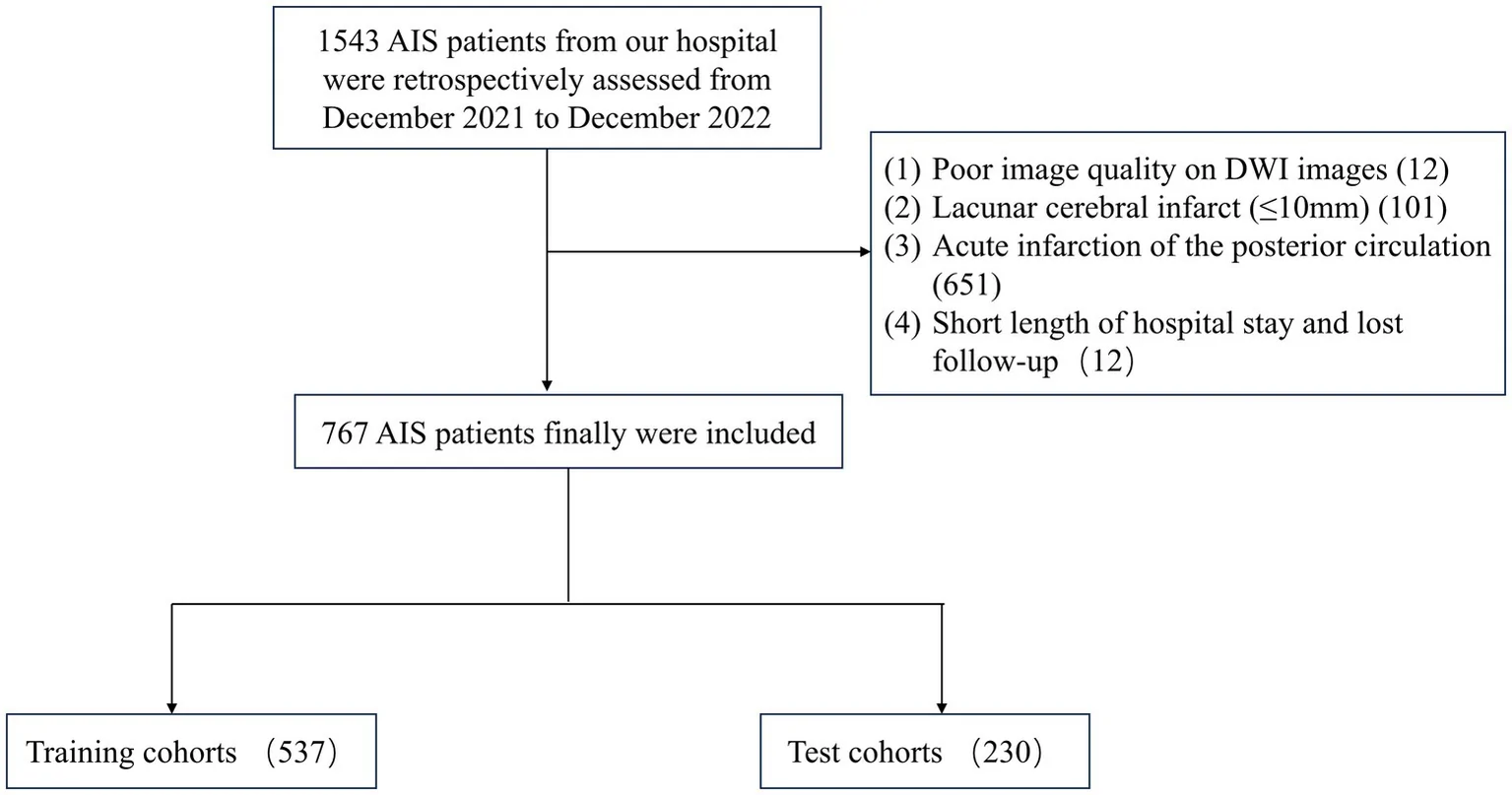
Distribution diagram of enrolled subjects in training and test cohorts.

In the retrospective cohorts, patients were diagnosed with acute ischemic stroke in accordance with AHA/ASA guidelines and admitted to Zhengzhou Central Hospital Affiliated to Zhengzhou University between December 2021 and December 2022. A total of 1,543 patients were initially screened, and 767 eligible patients were finally included after applying the predefined inclusion and exclusion criteria. The flow diagram of participant grouping into training and test cohorts is presented in Figure 1. The inclusion criteria were as follows: (1) age >18 years; (2) MRI performed within 3 days of stroke onset; (3) DWI-positive acute ischemic lesions. Exclusion criteria: (1) unqualified DWI image quality; (2) lacunar infarction with maximum diameter ≤10 mm; (3) isolated posterior circulation infarction; (4) hospital stay < 3 days or incomplete follow-up data missing ND assessment. As a primary clinical endpoint, neurological deterioration (ND) was defined as an increase of at least 4 points in the National Institutes of Health Stroke Scale (NIHSS score) between baseline and during hospitalization, including death during hospitalization. This approach aimed to capture all neurologic deterioration, including immediate, persistent worsening after admission or subsequent decline after initial improvement (18, 19).

This retrospective study was approved by the Ethics Committee of our hospital (No. ZXYY2024328), and the requirement for written informed consent was waived due to the anonymized retrospective data analysis. All research procedures strictly followed the standards stated in the Declaration of Helsinki.

### 2.2 Imaging protocol

The MRI-DWI images were obtained using three different MRI scanners at the Zhengzhou Central Hospital Affiliated to Zhengzhou University. The acquisition parameters were as follows: (1) Siemens: TR = 5,790 ms, TE = 73 ms, slice thickness 5 mm, slice spacing 2 mm, field of view 23 cm × 23 cm, matrix 150 × 150, echo gap 0.36 ms, b value 1,000 s/mm^2^; (2) General Electric (GE): TR = 2,738 ms, TE = 77 ms, slice thickness 6 mm, slice spacing 1.5 mm, field of view 24 cm ×  24 cm, matrix 384 × 320, excitation times 2, echo gap 0.8 ms, b value 1,000 s/mm^2^.

### 2.3 Dataset partitioning

To ensure robust generalization, all samples were randomly divided into training and test cohorts in a 7:3 ratio, with repeated iterations performed until no significant differences were observed in the clinical characteristics between the two cohorts (*p* > 0.05; Table 1). Hyperparameter tuning was conducted using a grid search strategy combined with five-fold cross-validation within the training set (Supplementary workflow). The identified optimal parameters were subsequently applied to the entire training cohort, and the model performance was evaluated on the independent test set.

**Table 1:** Baseline characteristics of the training and test cohorts.

Feature_name	All	Training cohort (537)	Test cohort (230)	*p*-value
Basic characteristics				
Age (Mean±SD)	65.46 ± 12.82	65.38 ± 13.00	65.65 ± 12.42	0.801
Admission NIHSS Score (Mean±SD)	5.19 ± 5.09	5.19 ± 5.35	5.18 ± 4.42	0.189
Gender (Percentile: %)				0.463
0	500 (65.19)	355 (66.11)	145 (63.04)	
1	267 (34.81)	182 (33.89)	85 (36.96)	
Laboratory test (Mean±SD)				
White Blood Cell Count	7.64 ± 2.69	7.63 ± 2.57	7.67 ± 2.95	0.981
Neutrophil Count	5.38 ± 2.58	5.37 ± 2.47	5.41 ± 2.83	0.868
Lymphocyte Count	1.72 ± 0.91	1.72 ± 0.83	1.72 ± 1.07	0.610
Red Blood Cells	4.56 ± 0.68	4.58 ± 0.68	4.52 ± 0.68	0.468
Hemoglobin	136.65 ± 20.94	137.47 ± 19.80	134.75 ± 23.30	0.231
Platelets	229.62 ± 75.47	232.02 ± 78.23	224.03 ± 68.41	0.172
CRP	6.26 ± 19.33	5.91 ± 17.08	7.09 ± 23.79	0.226
Creatinine	72.09 ± 34.94	71.39 ± 32.67	73.73 ± 39.75	0.595
BUN	6.17 ± 4.99	5.82 ± 3.07	7.01 ± 7.75	0.179
Uric Acid	322.90 ± 106.81	319.74 ± 105.34	330.29 ± 110.04	0.180
ALT	26.57 ± 20.95	26.29 ± 19.27	27.22 ± 24.45	0.826
AST	29.23 ± 28.58	28.12 ± 14.67	31.81 ± 47.10	0.864
Total Bilirubin	16.56 ± 9.62	16.58 ± 10.12	16.53 ± 8.34	0.911
Direct Bilirubin	5.39 ± 4.68	5.39 ± 4.78	5.37 ± 4.46	0.734
Indirect Bilirubin	10.94 ± 5.97	10.85 ± 5.59	11.14 ± 6.79	0.810
Glucose	8.34 ± 8.49	8.28 ± 9.66	8.49 ± 4.73	0.283
HbA1c	6.57 ± 1.63	6.51 ± 1.54	6.73 ± 1.83	0.161
Total Cholesterol	4.24 ± 1.12	4.22 ± 1.10	4.28 ± 1.15	0.640
Triglycerides	1.57 ± 1.44	1.58 ± 1.60	1.54 ± 0.95	0.499
HDL	1.06 ± 0.29	1.06 ± 0.30	1.05 ± 0.27	0.885
LDL	2.33 ± 0.82	2.31 ± 0.81	2.37 ± 0.85	0.429
TT3	0.77 ± 0.30	0.77 ± 0.34	0.77 ± 0.17	0.776
TT4	7.04 ± 1.31	7.04 ± 1.36	7.03 ± 1.18	0.741
FT3	2.44 ± 0.45	2.43 ± 0.47	2.46 ± 0.41	0.149
FT4	1.08 ± 1.02	1.10 ± 1.19	1.06 ± 0.43	0.749
TSH	2.04 ± 3.50	2.07 ± 3.99	1.97 ± 1.89	0.405
Homocysteine	19.38 ± 12.34	19.61 ± 10.97	18.83 ± 15.07	0.228
D-Dimer	1.00 ± 3.90	0.89 ± 2.09	1.26 ± 6.36	0.120
Medical history (Percentile: %)				
Hypertension				0.814
0	277 (36.11)	192 (35.75)	85 (36.96)	
1	490 (63.89)	345 (64.25)	145 (63.04)	
Diabetes				1.000
0	541 (70.53)	379 (70.58)	162 (70.43)	
1	226 (29.47)	158 (29.42)	68 (29.57)	
Coronary Heart_Disease				0.510
0	621 (80.96)	431 (80.26)	190 (82.61)	
1	146 (19.04)	106 (19.74)	40 (17.39)	
Cerebral Infarction				0.371
0	562 (73.27)	399 (74.30)	163 (70.87)	
1	205 (26.73)	138 (25.70)	67 (29.13)	
Intracerebral Hemorrhage				0.255
0	752 (98.04)	529 (98.51)	223 (96.96)	
1	15 (1.96)	8 (1.49)	7 (3.04)	
Atrial Fibrillation				0.922
0	721 (94.00)	504 (93.85)	217 (94.35)	
1	46 (6.00)	33 (6.15)	13 (5.65)	
Smoking				0.301
0	542 (70.66)	373 (69.46)	169 (73.48)	
1	225 (29.34)	164 (30.54)	61 (26.52)	
Alcohol consumption				1.000
0	641 (83.57)	449 (83.61)	192 (83.48)	
1	126 (16.43)	88 (16.39)	38 (16.52)	

SD, Standard deviation; NIHSS, National Institute of Health Stroke Scale; CRP, C-reactive protein; HDL, high-density lipoprotein cholesterol; LDL, low-density lipoprotein cholesterol; BUN, blood urea nitrogen; ALT, alanine aminotransferase; AST, aspartate aminotransferase; TT3, total triiodothyronine; TT4, total thyroxine; FT3, free triiodothyronine; FT4, free thyroxine; TSH, thyroid stimulating hormone.

### 2.4 Image preprocessing, ROI delineation and reproducibility verification

#### 2.4.1 Image preprocessing protocol

All brain MRI-DWI sequences were first converted to Neuroimaging Informatics Technology Initiative (NIfTI) format from original Digital Imaging and Communications in Medicine (DICOM) files. To normalize spatial resolution across different imaging equipment and scanning parameters, all images were resampled to an isotropic voxel size of 1 mm^3^ via spline interpolation. Global intensity normalization was further performed to eliminate signal heterogeneity caused by variable acquisition settings, ensuring stable and reproducible radiomic feature extraction for subsequent region of interest (ROI) analysis.

#### 2.4.2 ROI annotation and reproducibility assessment

Infarction core regions were manually delineated slice-by-slice on axial DWI cross-sectional images using ITK-SNAP software by two neurologists (Dr. Si and Dr. Zong). To quantitatively evaluate the reproducibility of manual segmentation, 50 patients were randomly selected from the full cohort for repeat delineation consistency testing. Dr. Zong (5 years of neurological imaging experience) re-segmented the 50 cases twice with a 2-week washout interval to calculate intra-observer ICC; Dr. Si (10 years of neurological imaging experience) completed one independent segmentation of the same 50 cases to calculate inter-observer ICC. Any inconsistent segmentation boundaries between the two raters were adjudicated by a senior neuroradiologist (Dr. Wang, over 20 years of experience) to finalize the ROI mask for subsequent feature extraction. ICC > 0.75 was defined as acceptable consistency. The ICC values of intra-observer and inter-observer reproducibility of imaging features were listed in Supplementary Table S1. The median intra-observer ICC was 0.921, while the median inter-observer ICC reached 0.895, indicating excellent inter- and intra-rater agreement for infarct ROI segmentation.

### 2.5 Peri-infarction region construction

**Figure 2:**
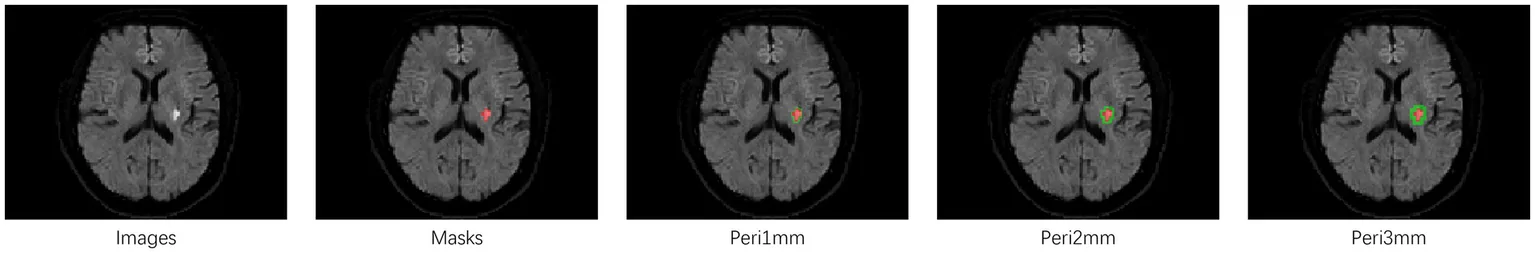
Generation of layered peri-infarct regions via stepwise morphological dilation.

To capture the peri-infarct microenvironment, we implemented a stepwise annular expansion strategy based on morphological dilation. The original infarction segmentation mask was expanded outward by 1, 2, and 3 mm, using the OnekeyAI mask-padding toolkit. At each expansion level, subtraction from the preceding inner boundary was performed to generate non-overlapping ring-shaped peri-infarction regions. This procedure ensured that the infarction core and adjacent peri-bands remained distinct while maintaining the structural continuity of the surrounding tissue. Feature extraction from these concentric peri-infarction layers allowed us to examine spatial gradients in the local microenvironment and assess how varying distances from the infarction margin influenced predictive performance (Figure 2).

### 2.6 Feature extraction

Radiomic features were organized into three major categories—morphological, intensity-based, and texture-based—to capture the full spectrum of the infarction phenotypes. Morphological descriptors reflect lesion geometry, such as shape, volume, and surface irregularity. Intensity-based features summarize voxel value distributions, whereas texture features quantify spatial heterogeneity using matrix-based approaches, including the gray-level co-occurrence matrix (GLCM), gray-level run length matrix (GLRLM), gray-level size zone matrix (GLSZM), and neighboring gray tone difference matrix (NGTDM).

Feature computation was performed at both the global and subregional levels. For global characterization, features were extracted from the entire infarction volume of interest. All features were extracted using PyRadiomics (v3.0.1) in compliance with the Imaging Biomarker Standardization Initiative (IBSI).

### 2.7 Feature selection

#### 2.7.1 Dataset partition and class imbalance processing

The total cohort included 767 AIS patients, with 85 ND-positive cases (11.08%). The cohort was randomly split into a 70% training set (*n* = 537) and 30% independent test set (*n* = 230) via stratified sampling to ensure consistent ND prevalence across subsets. To address class imbalance, Synthetic Minority Oversampling Technique (SMOTE) was applied exclusively to the training set to generate synthetic ND samples via k-nearest neighbor linear interpolation, balancing the ND-to-non-ND ratio to 1:1. No resampling was performed on the test set, which retained the original natural sample distribution to objectively evaluate model performance. SMOTE was implemented using the imbalanced-learn (imblearn, v0.11.0) Python library, with the number of nearest neighbors set to 5. The detailed sample distribution before and after SMOTE is provided in Supplementary Table S2.

#### 2.7.2 Multistage feature screening pipeline

All feature selection steps including t-tests, correlation filtering, the Minimum Redundancy Maximum Relevance (mRMR) and Least Absolute Shrinkage and Selection Operator (LASSO) regression were strictly conducted solely on the training cohort; the test set was never involved in any feature filtering or parameter optimization to avoid data leakage. All features underwent Z-score normalization to standardize scales. An initial univariate screening was performed with independent t-tests, retaining features significantly associated with the endpoint (*p* < 0.05). To control redundancy, highly correlated features (Pearson r > 0.9) were pruned by retaining the variable with higher variance.

Next, the mRMR algorithm was applied to identify the 32 most informative features, balancing relevance with non-redundancy. Finally, LASSO regression was used for embedded feature refinement, shrinking non-contributory variables toward zero. The optimal penalty parameter (*λ*) was determined via 10-fold cross-validation. This multi-stage procedure yielded a concise, non-redundant feature set optimized for predictive modeling.

### 2.8 Signature construction

Multiple predictive signatures were constructed to capture the distinct spatial and clinical aspects of infarction progression. All signatures followed a standardized feature selection pipeline and were trained using complementary machine learning classifiers to enhance their robustness and generalizability:

- *Radiomics signature (Rad)*: Derived from whole-infarction radiomic features across both time points, incorporating delta modeling to reflect temporal variations. Logistic Regression was applied to ensure interpretability, and Random Forest was used to capture nonlinear relationships.
- *Peritumoral signatures (Peri-xmm)*: Independent models were generated for peri-infarction expansions of 1, 2, and 3 mm, allowing the evaluation of spatial gradients in the microenvironment surrounding the infarction.
- *Clinical signature*: Constructed using the complete set of clinical variables, modeled with the same algorithms applied to the radiomics signature, to provide a direct comparison between clinical and imaging-based predictors.
- *Combined signature*: A multimodal model was built by integrating the most informative imaging features (radiomics and peri-infarction) with clinically significant variables identified through univariate and multivariate analyses (*p* < 0.05). To facilitate clinical application, a nomogram was developed for the intuitive visualization of the predictive model.

Based on DWI radiomics and clinical features, machine learning models were constructed for forecasting ND using five classifiers: Logistic Regression (LR), Random Forest (RF), Extremely Randomized Trees (ExtraTrees), eXtreme Gradient Boosting (XGBoost), and Light Gradient Boosting Machine (LightGBM) (Supplementary workflow). Model performance was evaluated using ROC curve analysis, with AUC as the primary indicator, sensitivity, specificity, accuracy, positive predictive value (PPV), and negative predictive value (NPV). Calibration was assessed using calibration plots alongside the Hosmer–Lemeshow (HL) test, while clinical benefit was quantified through DCA across varying decision thresholds.

### 2.9 Statistical analysis

This study was designed and reported in strict accordance with the Transparent Reporting of a multivariable prediction model for Individual Prognosis Or Diagnosis (TRIPOD) statement. The completed TRIPOD checklist is available as Supplementary Table S7. All statistical analyses were conducted on the OnekeyAI platform (v3.1.8) using Python 3.7.12. Categorical variables were compared using chi-square tests. For continuous variables, normality was first assessed with the Shapiro–Wilk test; normally distributed variables were compared with independent-samples t-tests, while non-normally distributed variables were compared with the Mann–Whitney U test. A two-sided *p* < 0.05 was considered statistically significant. Pairwise AUC comparisons among six models (Clinical, intra-infarction radiomics, Peri1/2/3 mm, Combined) were performed using the DeLong test for correlated ROC curves. This test calculated Z-statistics and two-sided *p*-values with covariance adjustment for intra-sample correlation, separately in training and test cohorts. AUC differences with *p* < 0.05 were regarded as statistically significant. To quantitatively compare calibration performance among all six predictive models, calibration slope and intercept were calculated for each model in both training and test cohorts, with the HL test conducted to assess global model fit, with all metrics summarized in Supplementary Table S8.

## 3 Results

### 3.1 Study workflow and baseline patient characteristics

**Figure 3:**
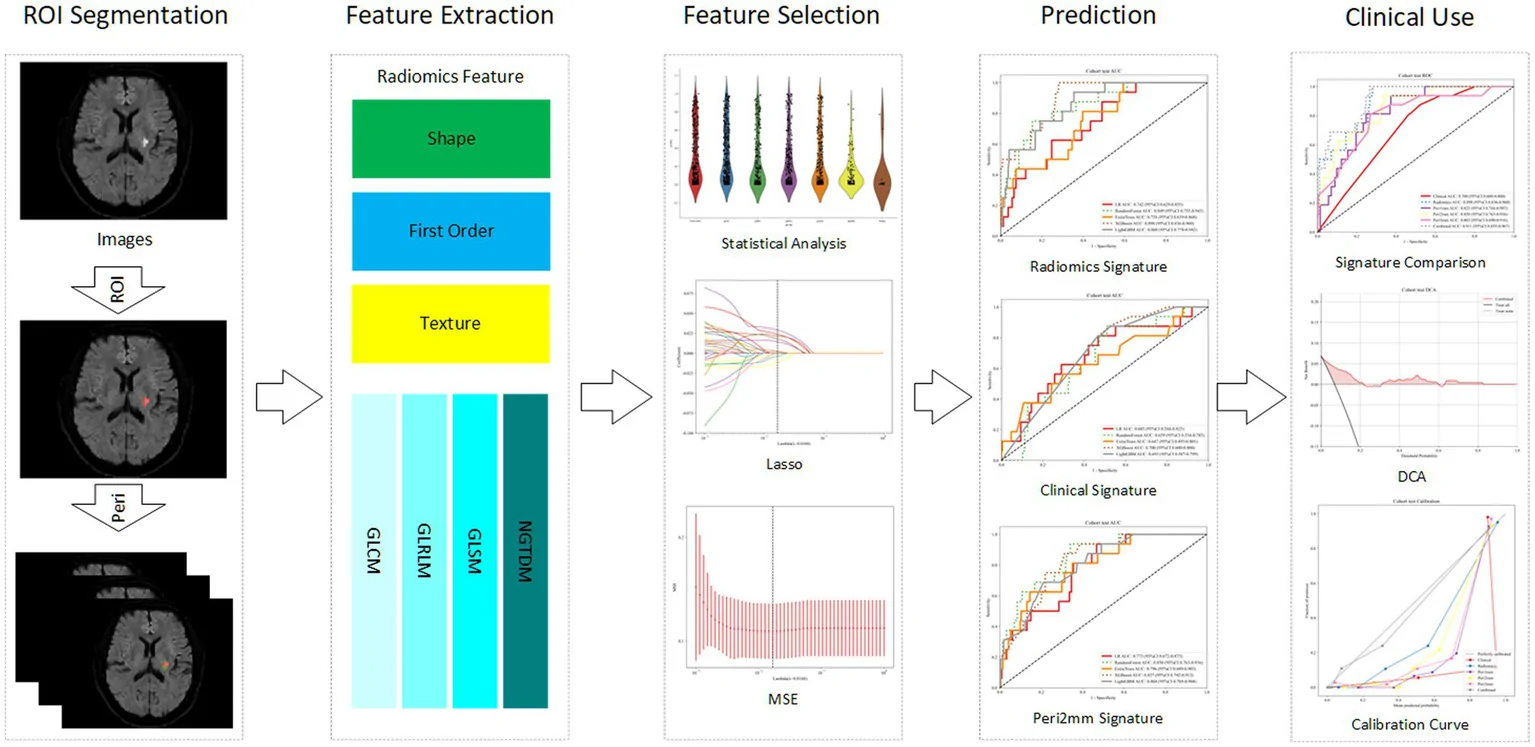
Overall workflow of the present study, covering sequential procedures including brain image segmentation, radiomic feature extraction, predictive model construction, and nomogram development.

The study flowchart includes image segmentation, feature extraction, model establishment, and a nomogram (Figure 3). In total, 767 patients were included in this study, including 500 men (65.19%) and 267 women (34.81%). Of these patients, 85 (11.08%) presented with ND. In total, 537 and 230 patients were included in the training and test cohorts, respectively. The baseline characteristics of the training and test cohorts are presented in Table 1. Basic variables of patients, showed no statistical differences (*p* > 0.05) between the training and test cohorts.

### 3.2 Analysis of clinical features and model development

We performed a comprehensive univariate analysis of all clinical variables and calculated OR and corresponding *p*-values for each predictor (Table 2). CRP [OR = 1.002; 95% confidence interval (CI), 1.001–1.004], AST (OR,=1.002; 95% CI, 1.001–1.004), Total Bilirubin (OR = 1.003; 95% CI, 1.001–1.005), Glucose (OR = 1.004; 95% CI, 1.002–1.007), Admission NIHSS Score (OR = 1.008; 95% CI, 1.003–1.012), BUN (OR = 1.012; 95% CI, 1.004–1.019), D-Dimer (OR = 1.018; 95% CI, 1.006–1.029) were risk factors associated with ND (*p* < 0.05) in the training and testing cohort, respectively, as shown in Table 2. Of note, LDL showed a marginal trend toward correlation with ND in univariate analysis (OR = 1.036, *p* = 0.051), but it did not reach the statistical significance threshold and was excluded from multivariate regression modeling. Variables with *p*-values below 0.05 were considered statistically significant and subsequently included in the construction of the multivariate clinical model. This selection ensured that only the relevant clinical factors with demonstrated univariate associations were used for further analysis. In the multivariate analysis, glucose level (OR = 1.003; 95% CI, 1.001–1.006; *p* < 0.05) was an independent predictor of ND development.

**Table 2:** Univariate and multivariate analysis of clinical features.

Feature name	Univariate analysis		Multivariate analysis	
	OR, 95% CI	*p-*value	OR, 95% CI	*p-*value
Basic characteristics				
Age	1.002, (1.000–1.004)	0.132		
Admission NIHSS Score	1.008, (1.003–1.012)	<0.05	1.005, (1.001–1.010)	0.059
Gender	1.022, (0.971–1.075)	0.477		
Laboratory test				
White Blood Cell Count	1.003, (0.993–1.012)	0.64		
Neutrophil Count	1.006, (0.997–1.016)	0.294		
Lymphocyte Count	0.971, (0.944–0.999)	0.086		
Red Blood Cells	1.001, (0.967–1.037)	0.970		
Hemoglobin	0.999, (0.998–1.000)	0.183		
Platelets	1.000, (1.000–1.000)	0.614		
CRP	1.002, (1.001–1.004)	<0.05	1.001, (0.999–1.002)	0.469
Creatinine	1.001, (1.000–1.001)	0.276		
BUN	1.012, (1.004–1.019)	<0.05	1.009, (1.001–1.017)	0.072
Uric Acid	1.000, (1.000–1.000)	0.499		
ALT	1.000, (0.999–1.001)	0.936		
AST	1.002, (1.001–1.004)	<0.05	1.001, (0.999–1.003)	0.483
Total Bilirubin	1.003, (1.001–1.005)	<0.05	1.002, (1.000–1.005)	0.126
Direct Bilirubin	1.000, (0.995–1.005)	0.967		
Indirect Bilirubin	1.001, (0.997–1.005)	0.747		
Glucose	1.004, (1.002–1.007)	<0.05	1.003, (1.001–1.006)	**<0.050**
HbA1c	1.001, (0.985–1.016)	0.930		
Total Cholesterol	1.016, (0.994–1.038)	0.237		
Triglycerides	0.986, (0.971–1.001)	0.119		
HDL	1.019, (0.941–1.105)	0.695		
LDL	1.036, (1.006–1.066)	0.051		
TT3	1.046, (0.974–1.122)	0.297		
TT4	0.992, (0.974–1.010)	0.451		
FT3	0.965, (0.917–1.015)	0.245		
FT4	1.008, (0.988–1.028)	0.529		
Homocysteine	0.998, (0.996–1.000)	0.115		
Medical history				
Hypertension	1.005, (0.957–1.057)	0.857		
Diabetes	1.015, (0.964–1.070)	0.632		
Coronary Heart Disease	1.016, (0.957–1.079)	0.656		
Cerebral Infarction	1.012, (0.959–1.069)	0.709		
Intracerebral_Hemorrhage	1.131, (0.930–1.377)	0.302		
Atrial_Fibrillation	0.992, (0.899–1.096)	0.898		
Smoking	0.956, (0.908–1.007)	0.156		
Alcohol_Consumption	1.009, (0.946–1.077)	0.810		

OR, odds ratio; 95% CI, 95% confidence interval. Bold values indicate statistically significant results (*p* < 0.05).

### 3.3 Extraction, selection of intra-infarction radiomic features and construction of radiomic signatures

**Figure 4:**
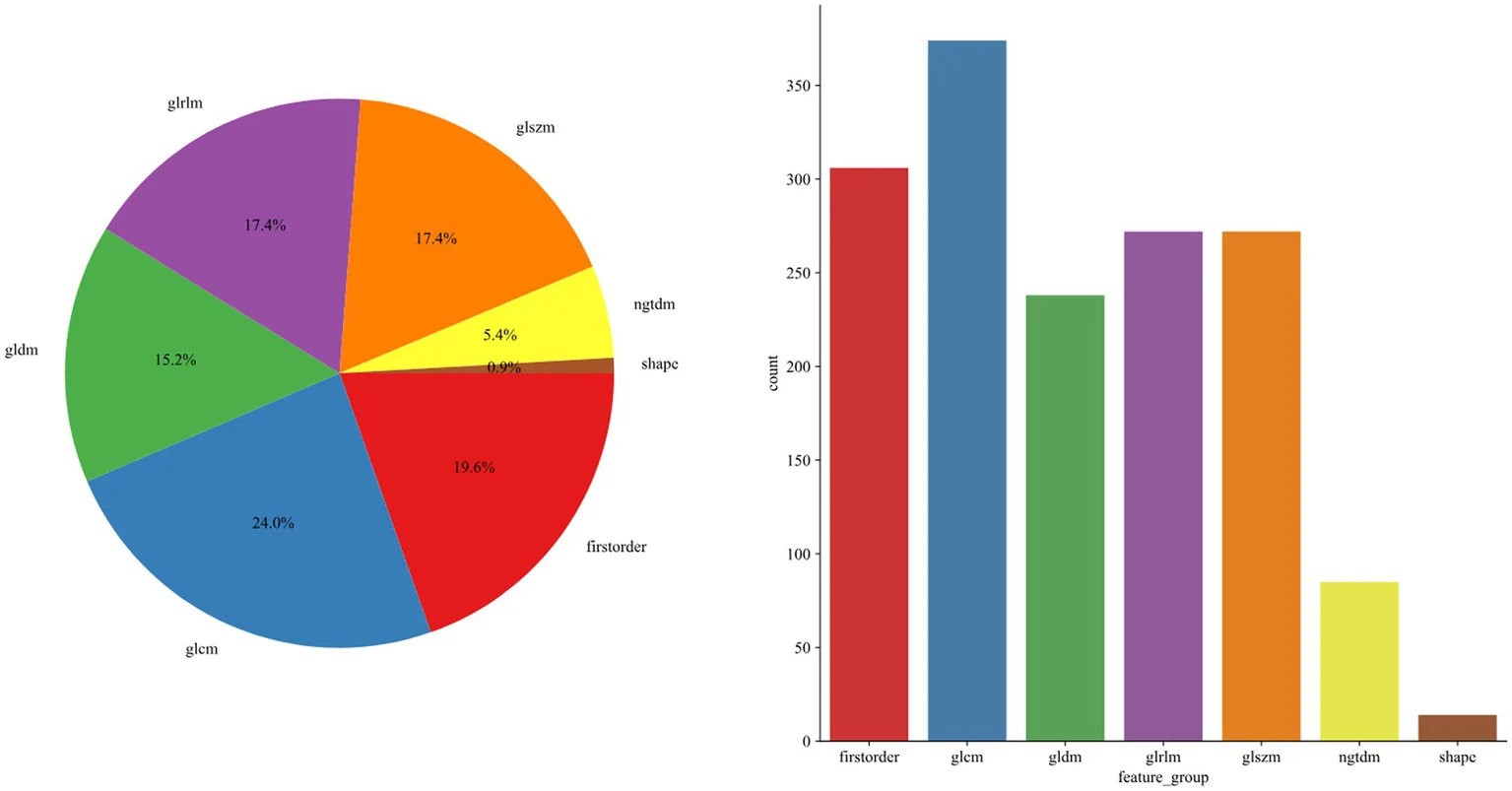
Composition proportion of all extracted intra-infarction radiomic features, categorized into first-order statistical features, shape features and multiple texture feature families.

**Figure 5:**
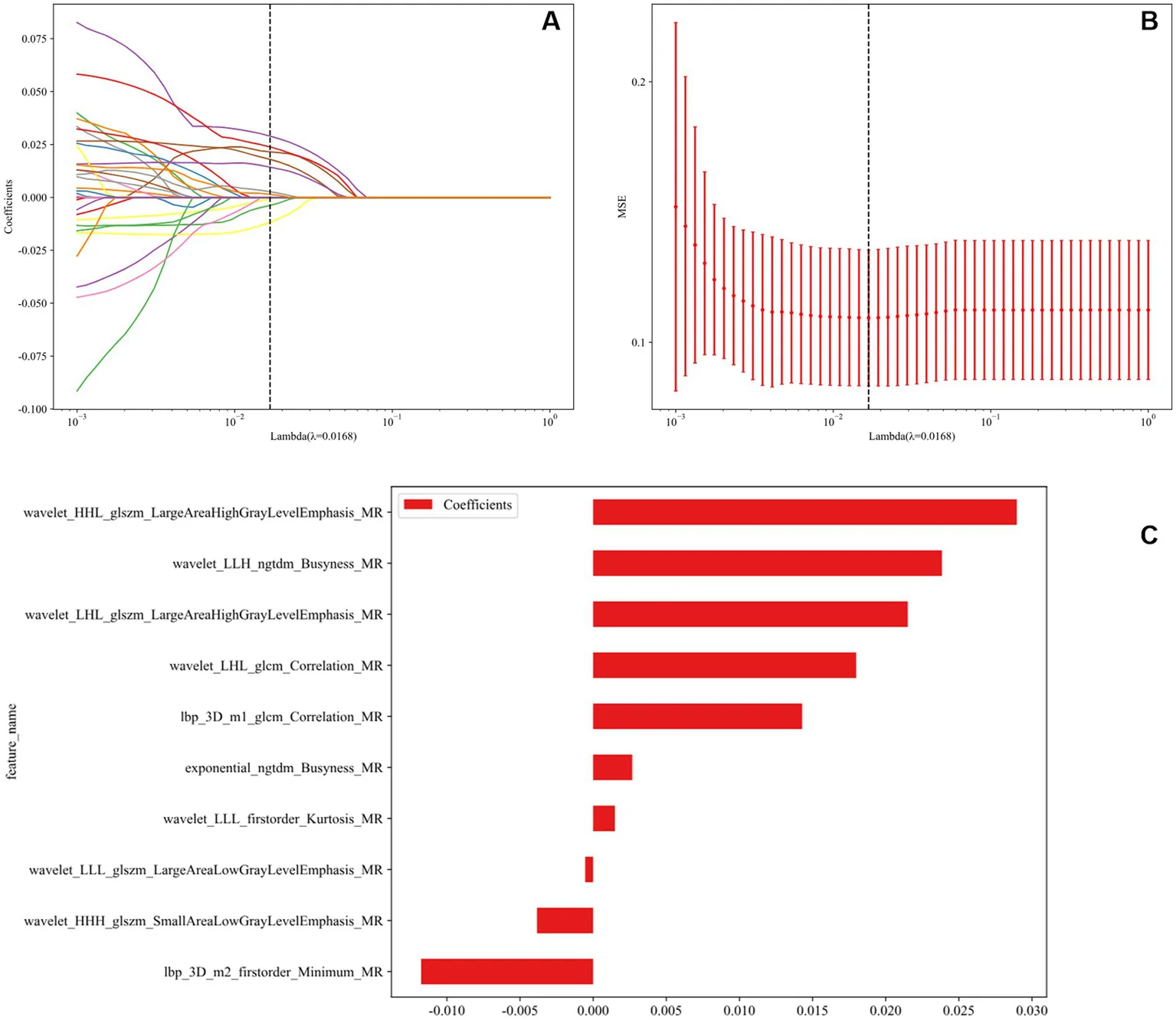
Feature screening results based on LASSO logistic regression. **(A)** The mean squared error (MSE) curve obtained via 10-fold cross-validation. **(B)** Tuning of the regularization parameter *λ* corresponding to the minimum MSE. **(C)** LASSO coefficient profiles of the 10 final selected intra-infarction radiomic features.

Our study extracted 1,561 handcrafted radiomic features from each region of interest and systematically organized them into three major categories: 14 shape descriptors reflecting infarction morphology, 306 first-order features quantifying voxel intensity distributions, and a comprehensive set of texture features designed to capture spatial heterogeneity patterns within the lesion (Figure 4). To isolate the most informative predictors while reducing overfitting, LASSO logistic regression was applied. A 10-fold cross-validation framework was used to optimize the regularization parameter (*λ*), with selection guided by minimizing the Mean Squared Error (MSE). Finally, we screened 10 features, including two first-order features and eight spatial heterogeneity features (Figure 5).

### 3.4 Evaluation metrics of intra-infarction radiomics models

**Figure 6:**
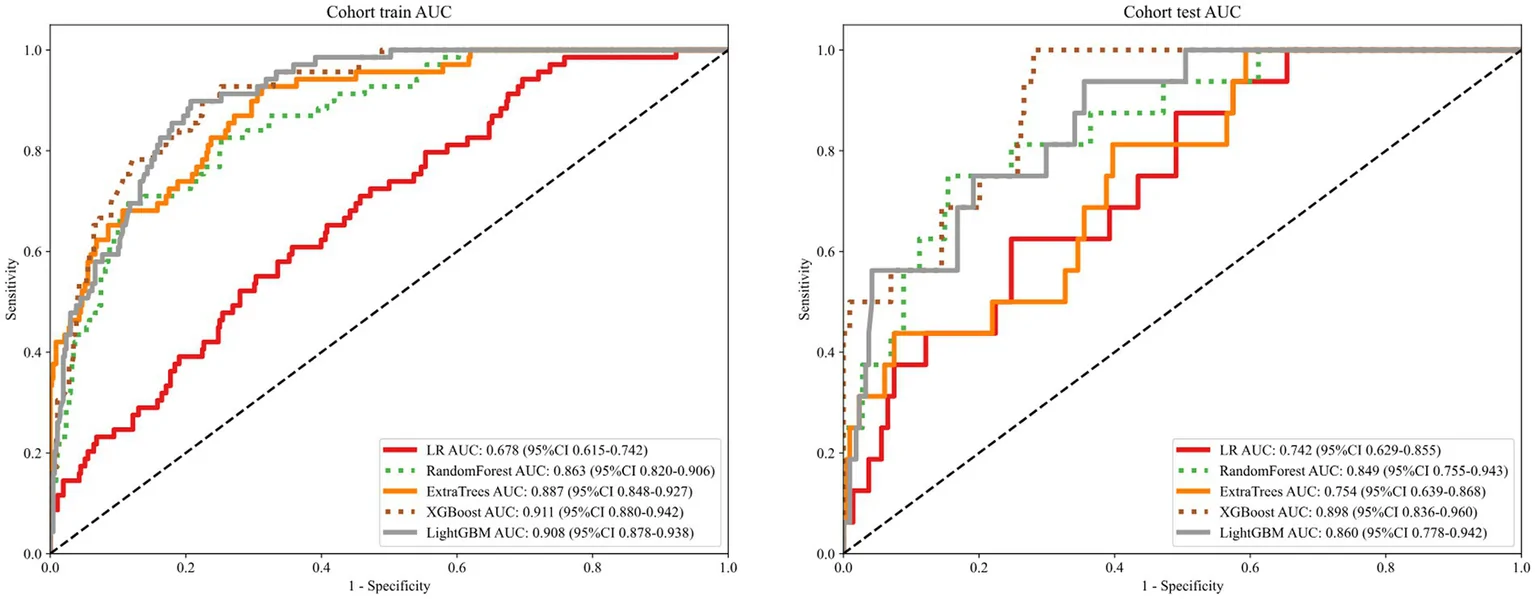
ROC curves of five different radiomics classifiers based on the intra-infarction signatures for ND prediction in training and test datasets.

Among the different classifiers, the XGBoost model demonstrated favorable balance between discrimination and robustness (Table 3; Figure 6). In the training cohort, the radiomics model achieved an AUC of 0.911 (95% CI: 0.880–0.942), a sensitivity of 0.982, a specificity of 0.748, an accuracy of 0.771, and an NPV of 0.986. In the test cohort, XGBoost maintained a consistent performance, yielding an accuracy of 0.739 and an AUC of 0.898 (95% CI: 0.836–0.960). The XGBoost classifier yielded a sensitivity point estimate of 1.000 (95% Wilson CI: 0.867–1.000) in the test cohort, accompanied by a specificity of 0.720 and an NPV of 1.000. The wide Wilson interval suggests considerable sampling variability due to the limited number of ND events.

**Table 3:** Predictive performance of multiple machine learning algorithms using intra-infarction radiomic signatures in training and test cohorts.

Model_name	Accuracy	AUC	95% CI	Sensitivity (95% Wilson CI:)	Specificity	PPV	NPV	Threshold	Cohort
LR	0.564	0.678	0.615–0.742	0.710 (0.585–0.809)	0.543	0.186	0.927	0.502	Training
LR	0.535	0.742	0.629–0.855	0.875 (0.694–0.956)_	0.509	0.118	0.982	0.468	Test
RandomForest	0.860	0.863	0.820–0.906	0.696 (0.571–0.798)	0.885	0.471	0.952	0.561	Training
RandomForest	0.839	0.849	0.755–0.943	0.750 (0.555–0.878)	0.846	0.267	0.978	0.561	Test
ExtraTrees	0.719	0.887	0.848–0.927	0.928 (0.834–0.971)	0.688	0.305	0.985	0.510	Training
ExtraTrees	0.617	0.754	0.639–0.868	0.812 (0.622–0.919)	0.603	0.133	0.977	0.497	Test
XGBoost	0.771	0.911	0.880–0.942	0.928 (0.834–0.971)	0.748	0.352	0.986	0.462	Training
XGBoost	0.739	0.898	0.836–0.960	1.000 (0.867–1.000)	0.720	0.211	1.000	0.470	Test
LightGBM	0.806	0.908	0.878–0.938	0.899 (0.797–0.953)	0.793	0.390	0.981	0.512	Training
LightGBM	0.665	0.860	0.778–0.942	0.937 (0.773–0.985)	0.645	0.165	0.993	0.435	Test

### 3.5 Signature comparison of peri-infarction models, clinical models and combined model

**Figure 7:**
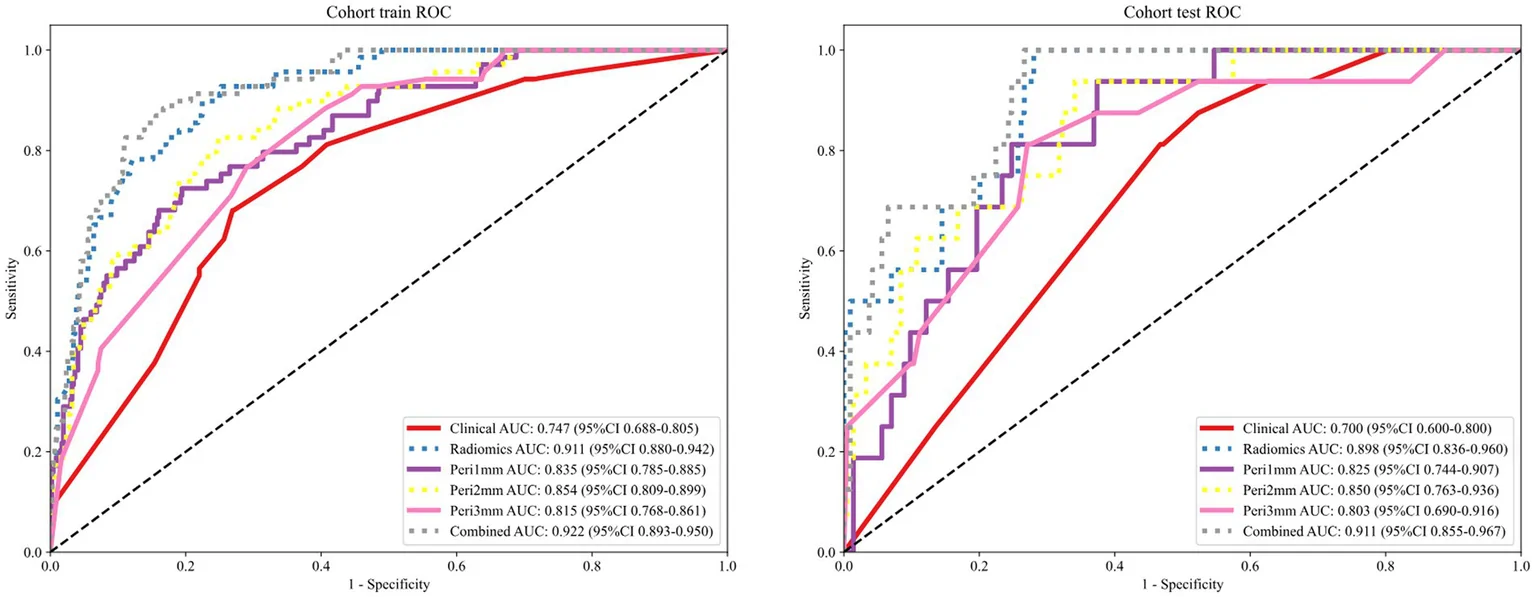
ROC curves of six predictive models for ND prediction in the training cohort, including clinical model, intra-infarction radiomics, three peri-infarction ring models (1 mm, 2 mm, 3 mm), and the combined clinical-radiomic model.

Additional details regarding Perixmm and clinical data are provided in Supplementary Materials A,B. In the training cohort, the intra-infarction model demonstrated superior predictive power, clearly outperforming the peri-infarction model. Among the peri-infarction regions, the 2 mm expansion achieved the highest discriminative ability (AUC = 0.854, 95% CI: 0.809–0.899) compared to the 1 mm (AUC = 0.835) and 3 mm (AUC = 0.815) expansions. The combined model, which integrated intra-infarction, peri-2 mm, and clinical features, further improved performance, reaching an AUC of 0.922 (95% CI: 0.893–0.950) with accuracy of 0.831. In the test cohort, intra-infarction radiomics maintained a strong performance (AUC = 0.898, sensitivity = 1.000, 95% Wilson CI:0.867–1.000), peri-2 mm showed the best results among the peri-infarction models (AUC = 0.850, sensitivity = 0.937), and the combined model achieved the highest overall performance with an AUC of 0.911 and a sensitivity point estimate of 1.000 (95% Wilson CI: 0.867–1.000; Table 4; Figure 7). These results highlight that radiomics features offered greater predictive value than peri-infarction signatures alone, though the peri-2 mm region consistently performed better than the 1 mm and 3 mm expansions. Notably, integrating intra-infarction radiomics with peri-2 mm features and clinical variables yielded the strongest overall performance, underscoring the complementary value of combining intra-infarction, peri-infarction, and clinical information to enhance prediction accuracy and generalizability.

**Table 4:** Predictive performance comparison of clinical, intra-infarction, peri-infarction and combined radiomic models in training and test cohorts.

Signature	Accuracy	AUC	95% CI	Sensitivity (95% Wilson CI:)	Specificity	PPV	NPV	Cohort
Clinical	0.724	0.747	0.689–0.805	0.681 (0.555–0.785)	0.731	0.272	0.940	Training
Radiomics	0.771	0.911	0.880–0.942	0.928 (0.834–0.971)	0.748	0.352	0.986	Training
Peri1mm	0.795	0.835	0.785–0.885	0.725 (0.601–0.822)	0.806	0.355	0.952	Training
Peri2mm	0.762	0.854	0.809–0.899	0.826 (0.711–0.901)	0.752	0.329	0.967	Training
Peri3mm	0.631	0.815	0.768–0.861	0.884 (0.779–0.943)	0.594	0.243	0.972	Training
Combined	0.831	0.922	0.893–0.950	0.899 (0.797–0.953)	0.821	0.425	0.982	Training
Clinical	0.504	0.700	0.600–0.800	0.875 (0.555–0.785)	0.477	0.111	0.981	Test
Radiomics	0.739	0.898	0.836–0.960	1.000 (0.867–1.000)	0.720	0.211	1.000	Test
Peri1mm	0.757	0.825	0.744–0.907	0.812 (0.622–0.919)	0.752	0.197	0.982	Test
Peri2mm	0.678	0.850	0.763–0.936	0.937 (0.773–0.985)	0.659	0.170	0.993	Test
Peri3mm	0.735	0.803	0.690–0.917	0.812 (0.622–0.919)	0.729	0.183	0.981	Test
Combined	0.752	0.911	0.855–0.967	1.000 (0.867–1.000)	0.734	0.219	1.000	Test

**Figure 8:**
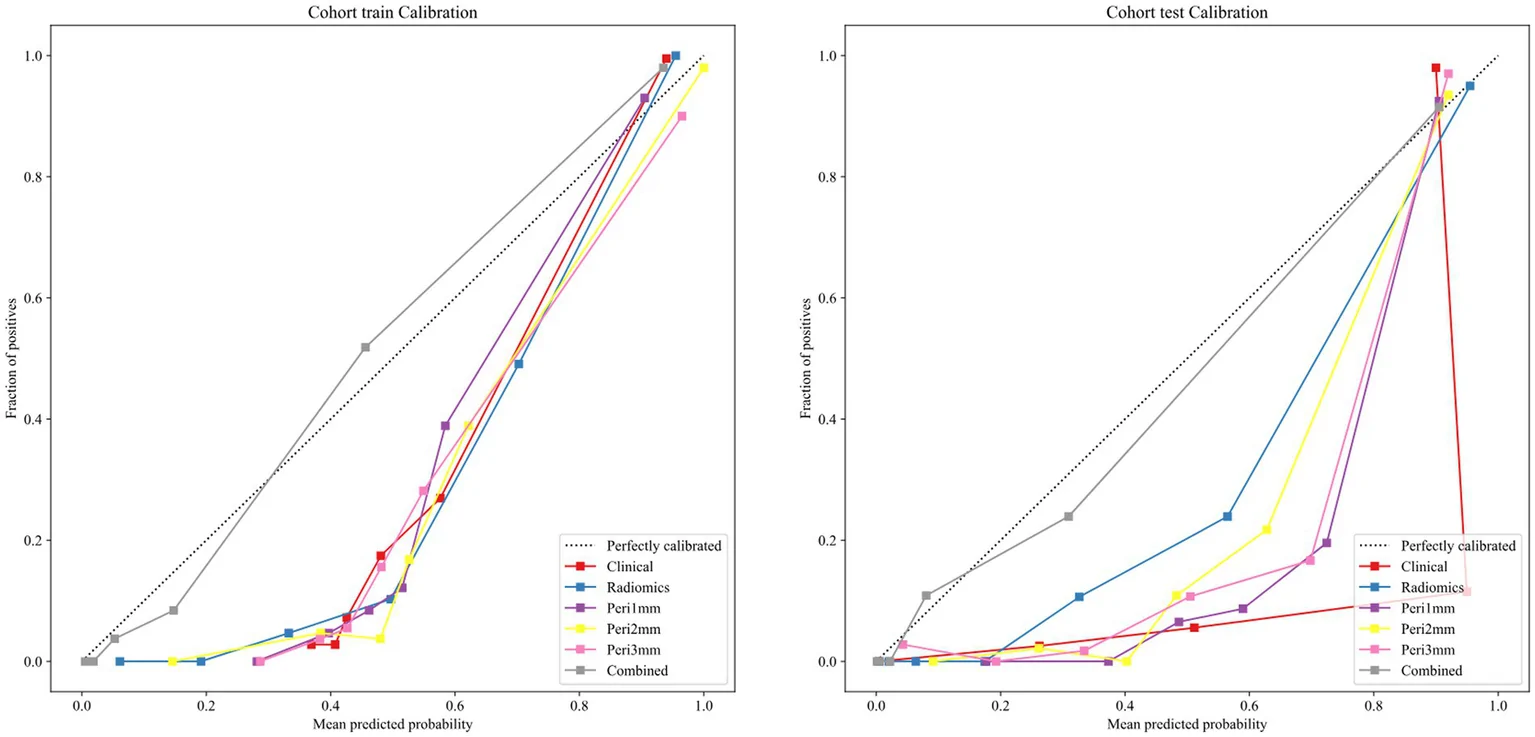
Calibration curves of all six predictive models in training and test datasets.

Calibration curves of all six predictive models were plotted in Figure 8 to visually compare their fitting performance in training and test cohorts. Quantitative calibration metrics including calibration slope, intercept and Hosmer–Lemeshow test *p*-values were calculated for every model and summarized in Supplementary Table S8. The calibration slope and calibration intercept provided complementary quantitative insights into calibration quality. The training and test cohorts yielded calibration slopes of 1.375 and 1.502, and calibration intercepts of 0.281 and −0.418 for the combined model, respectively. The HL test returned p-values of 0.536 (training cohort) and 0.329 (test cohort), both above the 0.05 significance threshold, indicating favorable consistency between predicted probabilities and observed clinical outcomes. As shown in Supplementary Table S8, all six models exhibited acceptable global calibration (all HL *p* > 0.05), with mild deviation from ideal calibration across all models. Stable calibration performance across both cohorts confirmed the robust generalizability of the combined model for clinical risk estimation.

**Figure 9:**
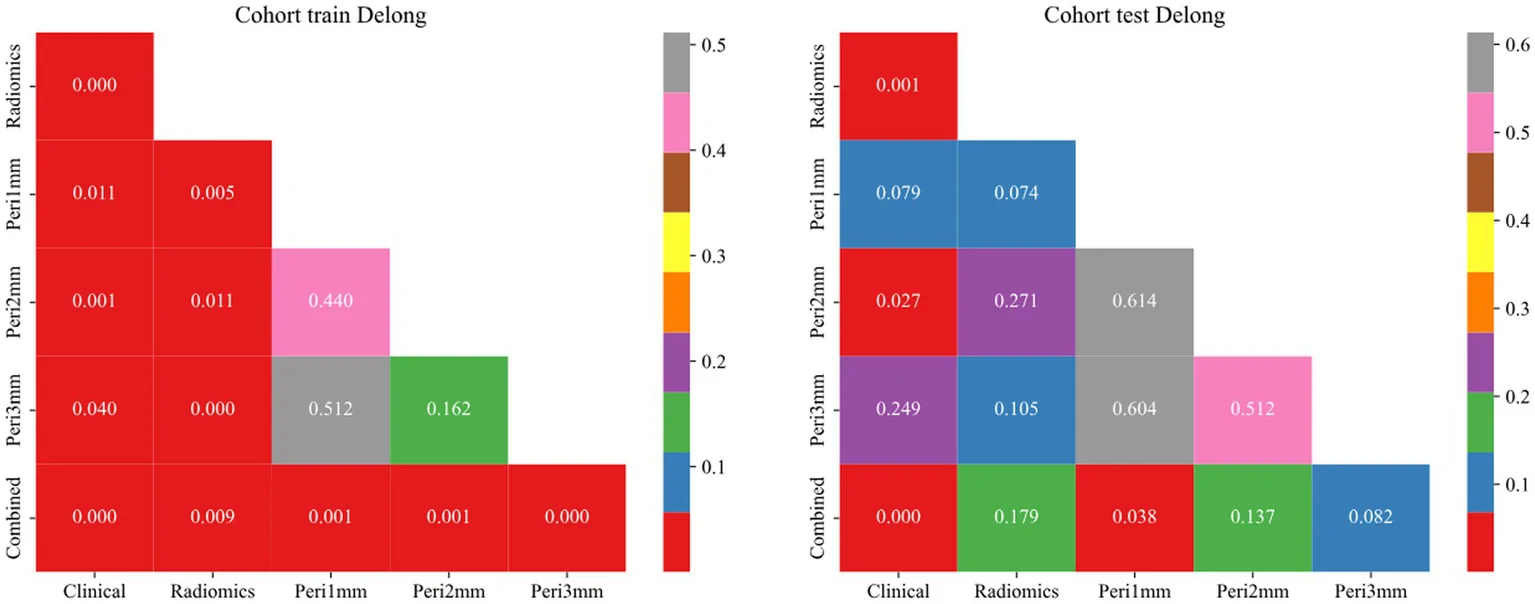
Results of pairwise DeLong test for AUC comparison of clinical, intra-infarction, three peri-infarction and combined prediction models in training and test datasets.

Pairwise AUC comparisons via DeLong test were conducted across all six predictive models in training and test cohorts (Figure 9). Within the training cohort, the combined multimodal model yielded significantly higher AUC than every single model (all *p* < 0.05). In the independent test cohort, the combined model exhibited numerically higher AUC values across all comparisons, yet statistical superiority was only detected against the clinical model (*p* = 0.000) and Peri1mm peri-infarct model (*p* = 0.038). No statistically meaningful AUC difference was observed when comparing the combined model to the core intra-infarction radiomics signature (*p* = 0.179), Peri2mm model (*p* = 0.137), or Peri3mm model (*p* = 0.082). These findings demonstrate that the discriminative gain of integrating peri-infarct imaging features and clinical variables was consistent internally in the training dataset, but could not be fully reproduced in the external test cohort.

### 3.6 Clinical use of models’ performance

**Figure 10:**
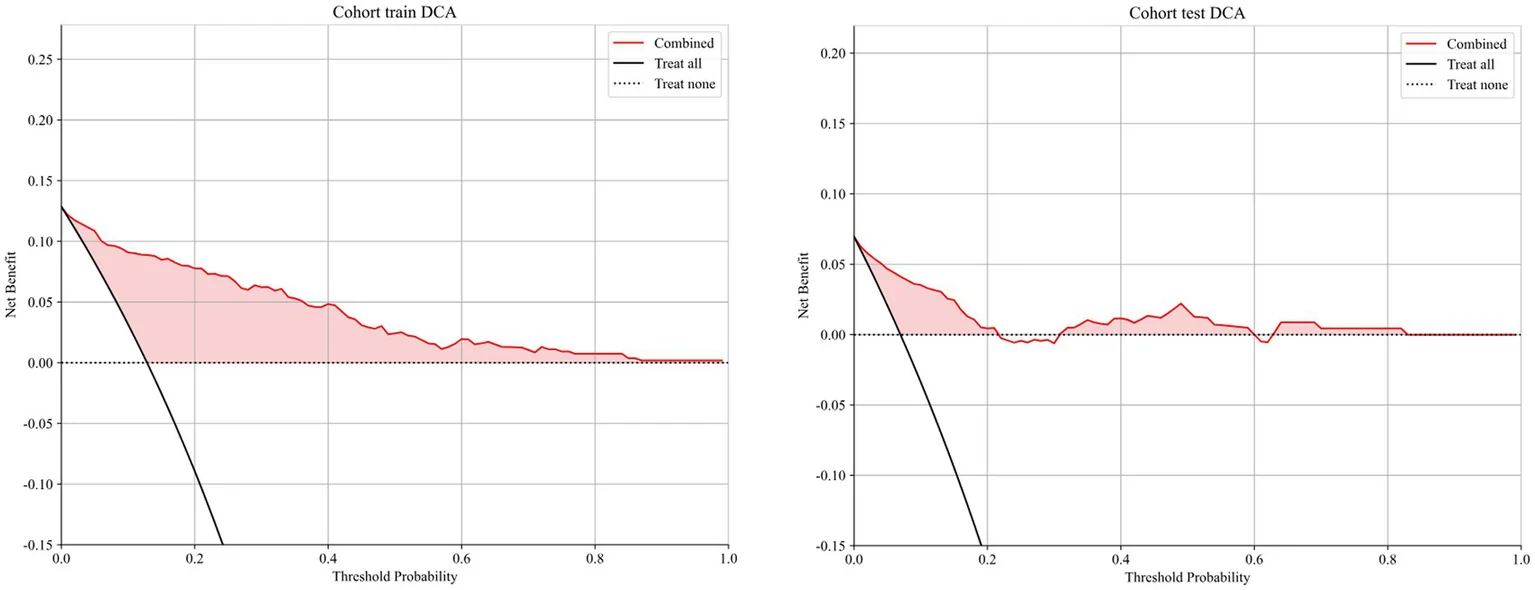
Decision curve analysis assessing clinical net benefit of the combined model in training and test datasets.

**Figure 11:**
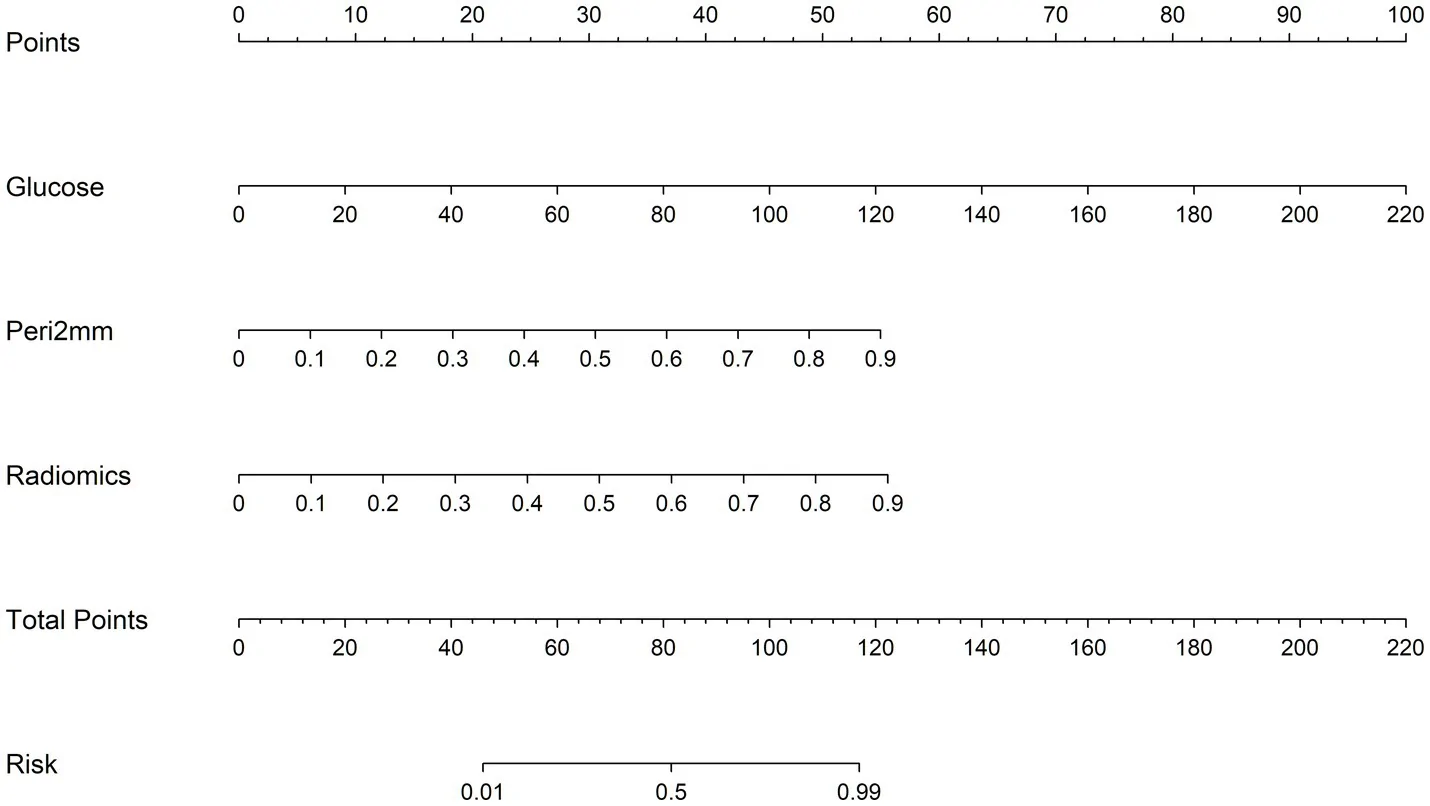
Nomogram for predicting ND risk in AIS patients integrating clinical and radiomic predictors.

The analysis of DCA indicates that our Combined model offers a notable advantage in terms of net benefit derived from predicted probabilities in both the training and testing cohorts (Figure 10). A nomogram was constructed to calculate an individual’s corresponding risk (Figure 11). A visual nomogram was constructed to quantitatively calculate the individualized risk of ND for each AIS patient. Each predictor is assigned a weighted score according to its predictive contribution. To operate the nomogram, vertical lines are drawn from the measured value of each variable to the uppermost “Points” axis to obtain the separate score for each feature. All three separate scores are summed to acquire the Total Points, and the total score is then mapped vertically downwards to the bottom Risk axis to read the corresponding probability of ND.

## 4 Discussion

In this study, MRI-based radiomics were systematically combined with peri-infarction analysis to establish predictive models for ND. Beyond the intra-infarction radiomics signature, we further assessed the peri-infarction regions by expanding the infarct boundaries at 1, 2, and 3 mm to investigate the added value of the surrounding microenvironment. A comparative evaluation revealed that intra-infarction radiomics alone provided a reasonable baseline, but the inclusion of peri-infarction features markedly enhanced the predictive ability. Among the different margins, the 2 mm peri-infarction model consistently outperformed its 1 mm and 3 mm counterparts, whereas the 1 mm model showed the least discriminative power. These findings indicate that the peri-infarction context carries complementary information to intra-infarction radiomics, and that a moderate extension, particularly at 2 mm, strikes a balance between capturing biologically informative cues and minimizing irrelevant backgrounds, thereby yielding the most robust predictive performance.

In this study, MRI-based radiomics were systematically combined with peri-infarction analysis to establish predictive models for ND. Beyond the intra-infarction radiomics signature, we further assessed the peri-infarction regions by expanding the infarct boundaries at 1, 2, and 3 mm to investigate the added value of the surrounding microenvironment. Multivariable analysis confirmed glucose as an independent clinical predictor of ND, yet the clinical model only achieved moderate discriminative performance. Intra-infarction radiomic features yielded robust prediction, and the XGBoost algorithm delivered the best performance among intra-infarction model. Among the different margins, the 2 mm peri-infarction model consistently outperformed its 1 mm and 3 mm counterparts, whereas the 1 mm model showed the least discriminative power, as this margin precisely captures the pathophysiologically critical ischemic penumbra between irreversibly infarcted tissue and normal brain. Our LASSO feature coefficients further support this view: the top retained features (GLSZM LAHGLE, NGTDM Busyness and GLCM Correlation) precisely quantify the necrosis, heterogeneous edema and diffusion gradients unique to this penumbral transition zone. The combined model integrating glucose, intra-infarction and peri-2 mm radiomic signatures achieved the highest AUC, favorable calibration and steady net clinical benefit, with perfect test-set sensitivity. DeLong tests revealed the combined model was statistically better than clinical and 1 mm peri-infarction models, without significant improvement over the intra-infarction radiomics model alone. Overall, peri-2 mm radiomics provides complementary pathological information to improve individualized ND risk stratification.

Consistent with the DeLong pairwise AUC comparisons, the predictive advantage of the combined multimodal model was fully statistically validated in the training cohort but incomplete in the test cohort. Although the combined model held numerically higher AUC than the core intra-infarction radiomics signature in the test set, no significant inter-model difference was observed (*p* = 0.179). This inconsistency between internal and external performance may be attributed to the limited sample size of the test cohort and single-center retrospective sampling bias, which reduced statistical power to detect subtle predictive gains brought by peri-infarction radiomic features. We caution against overinterpreting the 1.000 test-set sensitivity of the intra-infarction XGBoost and combined models. With only 25 ND events, its 95% Wilson CI (0.867–1.000) shows prominent sampling uncertainty. This high sensitivity from the Youden index cutoff comes at the cost of reduced PPV, requiring larger cohorts to confirm stable detection ability.

AIS remains a leading cause of long-term disability and mortality globally, with ND representing a critical barrier to favorable clinical outcomes. As a primary clinical endpoint, ND was defined as an increase of ≥4 points in total NIHSS during hospitalization. This standardized cutoff has been validated in large international stroke registries and randomized trial post-hoc analyses, where a ≥ 4-point NIHSS elevation represents functionally meaningful ischemic injury requiring intensified intervention (20, 21). Two alternative thresholds (NIHSS increment ≥1 or ≥2) are reported in previous reviews (22): A threshold of a 1-point rise is overly vulnerable to transient bedside assessment bias, while A threshold of a 2-point rise captures mild, often self-resolving neurological fluctuations with limited predictive value for infarct expansion and penumbral progression. Consistent with prior DWI radiomics studies focusing on ischemic microenvironment features, we adopted the ≥4-point definition to identify clinically relevant deterioration. Early neurological deterioration within 72 h occurs in 10–30% of AIS patients (22), while delayed neurological decline can also develop after72h even throughout hospitalization due to progressive peri-infarct microstructural injury (23); our endpoint incorporates all in-hospital ND events rather than only early worsening within 72 h.

Radiomics has emerged as a powerful tool to address this gap, enabling high-throughput extraction of quantitative features that reflect sub-visual tissue heterogeneity (13). Conventional clinical models only using baseline clinical factors showed modest predictive performance (internal AUC = 0.740, external AUC = 0.716), as they cannot reflect microstructural penumbral changes on DWI (24). Existing DWI radiomic models segmented infarct core without stratified peri-infarct tissue. Wang et al. built a pontine infarction nomogram (training AUC = 0.966, validation AUC = 0.920) but lacked layered peri-infarct analysis (21); Yu et al. established a perforator stroke multimodal radiomics model with validation AUC = 0.824, failing to separate core and peri-infarct rings (25). Neither study quantified the gradient pathological alterations in peri-lesional penumbra. Different from prior work, we created 1, 2, 3 mm annular peri-infarct masks and confirmed the 2 mm ring had optimal predictive value. Our integrated model combining intra-infarction radiomics, 2 mm peri-infarction signature and admission glucose attained AUCs of 0.922 (training) and 0.911 (test), with perfect test sensitivity, well-calibrated curves and stable clinical net benefit via DCA. Moreover, our model only relies on single DWI sequence without extra multi-modal scans, which is more clinically feasible. Notably, deep learning and transformer-based imaging models have emerged as promising approaches for AIS outcome prediction (26). We will explore deep learning and transformer architectures in future large-scale multi-center studies to further optimize ND prediction performance.

Notably, several statistically significant clinical predictors (including glucose) had OR values very close to 1.000, as these estimates represent the risk change per 1-unit increase in the continuous variable. The per-unit effect size is numerically small, and the corresponding clinical significance should be interpreted with caution. Despite the modest per-mmol/L effect, elevated admission glucose remains a biologically meaningful driver of ND, and its synergistic effect with radiomic features has clear pathological grounds (27, 28). Hyperglycemia disrupts endothelial tight junctions and the blood–brain barrier, accelerates cytotoxic edema within the infarct core, and amplifies heterogeneous inflammatory edema in ischemic penumbra; such subtle microstructural variations cannot be quantified by single laboratory glucose measurements alone, but can be sensitively captured by the radiological texture (29, 30).

The clinical utility of our combined model is further supported by its robust calibration and DCA, which confirmed net benefit across a range of threshold probabilities. The nomogram derived from this model—incorporating glucose, intra-infarction and Peri2mm radiomics—provides a user-friendly tool for clinicians to stratify ND risk. For instance, patients with a total nomogram score corresponding to 50% ND risk could be prioritized for hourly neurological monitoring or targeted interventions. However, the combined model has a low PPV of 0.219 in the test cohort, indicating around 80% of screened high-risk individuals are false positives. Therefore, this nomogram is more suitable as an initial screening tool rather than an independent diagnostic standard. Practical deployment of this radiomics model in routine stroke workflows remains challenging due to time-consuming manual infarct segmentation and hardware-intensive data analysis. The model can be embedded in PACS as a decision-support tool, and automated segmentation is expected to alleviate this burden in future.

Despite these strengths, this study has certain limitations. First, given that this study adopts a retrospective single-center design with merely internal train-test splitting and absent independent multi-center external validation, selection bias may have been introduced. Multi-center data with diverse populations and varying imaging protocols are essential to assess model stability. Large prospective multi-center collaborations are needed to externally validate and refine the model for broader clinical use. Second, manual ROI segmentation by radiologists may lead to inter-observer variability. Though ICC analysis confirmed high consistency, future work should validate automated segmentation to improve reproducibility. Third, patients with lacunar infarction and posterior circulation stroke were excluded, and ND was defined as NIHSS ≥4, which restricts the model’s generalizability. Future studies will incorporate these patient subgroups and adopt stratified NIHSS criteria (NIHSS ≥1 and NIHSS ≥2) to extend the model’s applicable scope. Finally, our model lacks histopathological validation given unfeasible invasive sampling in AIS patients. Still, radiomic features correlated with ND-related edema and necrosis indirectly support its clinical plausibility.

## 5 Conclusion

In conclusion, a DWI-based radiomics model combining intra-infarction, 2 mm peri-infarction and clinical features was established to predict neurological deterioration in acute ischemic stroke. The 2 mm peri-infarction band achieved the best performance among layered perilesional regions. The combined model yielded the highest discrimination and favorable calibration, though it’s incremental advantage over the intra-infarct-only model was not statistically significant in the test set. This routine DWI-based tool enables ND risk stratification, and prospective multicenter validation with automated segmentation is warranted for clinical translation.

## Data Availability

The original contributions presented in the study are included in the article/Supplementary material, further inquiries can be directed to the corresponding author/s.

## Glossary

SD: Standard deviation
NIHSS: National Institute of Health Stroke Scale
CRP: C-reactive protein
HDL: High-density lipoprotein cholesterol
LDL: Low-density lipoprotein cholesterol
BUN: Blood Urea Nitrogen
ALT: Alanine aminotransferase
AST: Aspartate aminotransferase
TT3: Total triiodothyronine
TT4: Total thyroxine
FT3: Free triiodothyronine
FT4: Free thyroxine
TSH: Thyroid stimulating hormone
OR: Odds ratio
95% CI: 95% confidence interval
AIS: Acute ischemic stroke
ND: Neurological deterioration
DWI: Diffusion-weighted imaging
MRI: Magnetic resonance imaging
AUC: Area under the receiver operating characteristic curve
ROC: Receiver operating characteristic
DCA: Decision curve analysis
ICC: Intraclass correlation coefficient
ROI: Region of interest
NIfTI: Neuroimaging Informatics Technology Initiative
DICOM: Digital Imaging and Communications in Medicine
GLCM: Gray-level co-occurrence matrix
GLRLM: Gray-level run length matrix
GLSZM: Gray-level size zone matrix
NGTDM: Neighboring gray tone difference matrix
IBSI: Imaging Biomarker Standardization Initiative
SMOTE: Synthetic Minority Oversampling Technique
mRMR: Minimum Redundancy Maximum Relevance
LASSO: Least Absolute Shrinkage and Selection Operator
LR: Logistic Regression
RF: Random Forest
ExtraTrees: Extremely Randomized Trees
XGBoost: eXtreme Gradient Boosting
LightGBM: Light Gradient Boosting Machine
HL: Hosmer–Lemeshow test
TRIPOD: Transparent Reporting of a multivariable prediction model for Individual Prognosis or Diagnosis
PPV: Positive predictive value
NPV: Negative predictive value
MSE: Mean Squared Error
HbA1c: glycated hemoglobin
PACS: Picture Archiving and Communication Systems
GE: General Electric
TR: Repetition time
TE: Echo time
AHA/ASA: American Heart Association/American Stroke Association
Wilson CI: Wilson confidence interval
ADC: Apparent diffusion coefficient
FLAIR: Fluid-attenuated inversion recovery
