# Supplementary material for: Development and validation of a DWI-based intra- and peri-infarction radiomics model for predicting neurological deterioration in acute ischemic stroke
Source: Front Neurol. 2026 Aug 10;17:1825520. doi: 10.3389/fneur.2026.1825520 (PMC13500267; doi:10.3389/fneur.2026.1825520)
Supplement: Supplementary file 1 [file Table_1.docx]

**Supplementary Materials**

### Supplementary workflow: Supplement detailed machine learning workflow information

**1. Hyperparameter Settings**

All classifiers used in this study were tuned via grid search combined with five-fold cross-validation. The final optimal hyperparameter settings for each model are as follows:

| Model | Hyperparameters |
| --- | --- |
| Logistic Regression | penalty = 'none', max_iter = 8, C = 1/5 |
| LightGBM | n_estimators = 51, max_depth = 3, random_state = 0 |
| Random Forest | n_estimators = 99, max_depth = 4, min_samples_split = 4, random_state = 0 |
| Extra Trees | n_estimators = 66, max_depth = 9, min_samples_split = 2, random_state = 0 |
| XGBoost | n_estimators = 19, objective = 'binary:logistic', max_depth = 3, min_child_weight = 2, eval_metric = 'error' |

**2. Rationale for Classifier Selection**

We selected the above five classifiers for the following reasons: (1) Logistic Regression serves as a linear baseline model with good interpretability; (2) XGBoost, LightGBM, Random Forest, and Extra Trees are all ensemble learning methods based on decision trees, capable of automatically capturing non-linear interactions among features, showing good adaptability to high-dimensional radiomic data, and have been widely validated for their predictive performance in practical applications; (3) the comparison across diverse models helps verify the robustness of the results.

**3. Reproducibility Settings**

To ensure reproducibility, all algorithms requiring randomness were set with a fixed random seed (random_state = 0), and all experiments were conducted under the same random seed.

**Table S1. Intra-observer and inter-observer reproducibility of imaging feature**

| **Feature** | **Intraclass Correlation Coefficient (95% CI)** | |
| --- | --- | --- |
|  | **Intra-observer** | **Inter-observer** |
| Infarct core volume | 0.921 (0.872-0.968) | 0.895 (0.836-0.951) |
| Maximum diameter of infarct | 0.935 (0.891-0.972) | 0.902 (0.854-0.947) |
| Surface area of infarct | 0.918 (0.865-0.962) | 0.887 (0.829-0.942) |
| Peri-infarct 1mm region volume | 0.915 (0.868-0.961) | 0.882 (0.827-0.937) |
| Peri-infarct 2mm region volume | 0.927 (0.883-0.969) | 0.908 (0.861-0.953) |
| Peri-infarct 3mm region volume | 0.932 (0.891-0.972) | 0.915 (0.872-0.958) |

CI: Confidence interval

**Table S2.1 Sample Distribution of Original Training Set (70% Stratified Random Split)**

| **Sample Type** | **Sample Size** |
| --- | --- |
| Total training set sample size | 537 |
| ND-positive samples | 60 |
| ND-negative samples | 477 |
| Original ND positive rate | 11.17% |

**Table S2.2	Sample Distribution of Training Set After SMOTE Balancing**

| **Sample Type** | **Sample Size** |
| --- | --- |
| Total training set sample size (after balancing) | 954 |
| ND-positive samples (original + synthetic) | 477 |
| ND-negative samples (unchanged) | 477 |
| ND:non-ND ratio after balancing | 1:01 |
| ND positive rate after balancing | 50.00% |

### 3 Supplementary Materials: Peri-infarction 1, 2, 3 mm radiomic signatures and clinical model

### Details of 1mm Peri-infarction radiomic signatures

1C

1A

1B

Figure S1.Feature screening results based on LASSO logistic regression for 1mm Peri-infarction radiomics signature . (1A) Coefficient profiles generated from 10-fold cross-validation. (1B) Mean squared error (MSE) curve obtained via 10-fold cross-validation. (1C) Histogram of intratumor heterogeneity derived from the finally selected 1mm Peri-infarction radiomic features.

Tbale S3. Quantitative performance metrics of five radiomic models constructed using 1mm peri-infarction signature in the training and test cohorts.

| **model_name** | **Accuracy** | **AUC** | **95% CI** | **Sensitivity** | **Specificity** | **PPV** | **NPV** | **Cohort** |
| --- | --- | --- | --- | --- | --- | --- | --- | --- |
| LR | 0.728 | 0.830 | 0.781–0.878 | 0.797 | 0.718 | 0.294 | 0.960 | train |
| LR | 0.543 | 0.603 | 0.466– 0.739 | 0.750 | 0.528 | 0.106 | 0.966 | test |
| RandomForest | 0.829 | 0.910 | 0.874–0.946 | 0.884 | 0.821 | 0.421 | 0.980 | train |
| RandomForest | 0.591 | 0.722 | 0.613–0.832 | 0.875 | 0.570 | 0.132 | 0.984 | test |
| ExtraTrees | 0.795 | 0.835 | 0.785–0.885 | 0.725 | 0.806 | 0.355 | 0.952 | train |
| ExtraTrees | 0.757 | 0.825 | 0.744– 0.907 | 0.812 | 0.752 | 0.197 | 0.982 | test |
| XGBoost | 0.693 | 0.806 | 0.752– 0.860 | 0.768 | 0.682 | 0.262 | 0.952 | train |
| XGBoost | 0.604 | 0.742 | 0.638– 0.846 | 0.875 | 0.584 | 0.136 | 0.984 | test |
| LightGBM | 0.767 | 0.835 | 0.787– 0.883 | 0.812 | 0.761 | 0.333 | 0.965 | train |
| LightGBM | 0.483 | 0.757 | 0.655–0.859 | 1.000 | 0.444 | 0.119 | 1.000 | test |

Figure S2.ROC curves of five different radiomics classifiers based on 1mm peri-infarction signature for prediction in training and test datasets.

### Details of 2mm Peri-infarction radiomic signatures

3C

3B

3A

Figure S3.Feature screening results based on LASSO logistic regression for 2mm Peri-infarction radiomics signature . (3A): Coefficient profiles generated from 10-fold cross-validation. (3B): Mean squared error (MSE) curve obtained via 10-fold cross-validation. (3C): Histogram of intratumor heterogeneity derived from the finally selected 1mm Peri-infarction radiomic features.

Tbale S4. Quantitative performance metrics of five radiomic models constructed using 2mm peri-infarction signature in the training and test cohorts.

| **model_name** | **Accuracy** | **AUC** | **95% CI** | **Sensitivity** | **Specificity** | **PPV** | **NPV** | **Cohort** |
| --- | --- | --- | --- | --- | --- | --- | --- | --- |
| LR | 0.669 | 0.776 | 0.723–0.829 | 0.826 | 0.645 | 0.256 | 0.962 | train |
| LR | 0.561 | 0.773 | 0.672–0.875 | 0.937 | 0.533 | 0.130 | 0.991 | test |
| RandomForest | 0.762 | 0.854 | 0.809–0.899 | 0.826 | 0.752 | 0.329 | 0.967 | train |
| RandomForest | 0.678 | 0.850 | 0.763–0.936 | 0.937 | 0.659 | 0.170 | 0.993 | test |
| ExtraTrees | 0.741 | 0.841 | 0.796–0.885 | 0.841 | 0.726 | 0.312 | 0.969 | train |
| ExtraTrees | 0.839 | 0.796 | 0.689–0.903 | 0.625 | 0.855 | 0.244 | 0.968 | test |
| XGBoost | 0.806 | 0.875 | 0.836–0.915 | 0.797 | 0.808 | 0.379 | 0.964 | train |
| XGBoost | 0.700 | 0.827 | 0.742–0.912 | 0.875 | 0.687 | 0.173 | 0.987 | test |
| LightGBM | 0.773 | 0.844 | 0.797–0.892 | 0.841 | 0.763 | 0.343 | 0.970 | train |
| LightGBM | 0.783 | 0.804 | 0.705–0.904 | 0.687 | 0.790 | 0.196 | 0.971 | test |

Figure S4.ROC curves of five different radiomics classifiers based on 2mm peri-infarction signature for prediction in training and test datasets.

### Details of 3mm Peri-infarction radiomic signatures

5C

5A

5B

Figure S5.Feature screening results based on LASSO logistic regression for 3mm Peri-infarction radiomics signature . (5A): Coefficient profiles generated from 10-fold cross-validation. (5B): Mean squared error (MSE) curve obtained via 10-fold cross-validation. (5C): Histogram of intratumor heterogeneity derived from the finally selected 1mm Peri-infarction radiomic features.

Tbale S5. Quantitative performance metrics of five radiomic models constructed using 3mm peri-infarction signature in the training and test cohorts.

| **model_name** | **Accuracy** | **AUC** | **95% CI** | **Sensitivity** | **Specificity** | **PPV** | **NPV** | **Cohort** |
| --- | --- | --- | --- | --- | --- | --- | --- | --- |
| LR | 0.715 | 0.775 | 0.721–0.830 | 0.681 | 0.720 | 0.264 | 0.939 | train |
| LR | 0.904 | 0.695 | 0.544–0.846 | 0.437 | 0.939 | 0.350 | 0.957 | test |
| RandomForest | 0.696 | 0.853 | 0.812–0.894 | 0.899 | 0.667 | 0.284 | 0.978 | train |
| RandomForest | 0.565 | 0.785 | 0.681–0.890 | 0.937 | 0.537 | 0.132 | 0.991 | test |
| ExtraTrees | 0.674 | 0.777 | 0.725–0.829 | 0.768 | 0.660 | 0.250 | 0.951 | train |
| ExtraTrees | 0.622 | 0.745 | 0.625– 0.864 | 0.875 | 0.603 | 0.141 | 0.985 | test |
| XGBoost | 0.631 | 0.815 | 0.768–0.861 | 0.884 | 0.594 | 0.243 | 0.972 | train |
| XGBoost | 0.735 | 0.803 | 0.690–0.916 | 0.812 | 0.729 | 0.183 | 0.981 | test |
| LightGBM | 0.728 | 0.859 | 0.819–0.899 | 0.899 | 0.703 | 0.308 | 0.979 | train |
| LightGBM | 0.570 | 0.788 | 0.675–0.900 | 0.937 | 0.542 | 0.133 | 0.991 | test |

Figure S6.ROC curves of five different radiomics classifiers based on 3mm peri-infarction signature for prediction in training and test datasets.

**Details of clinical radiomic signatures**

Tabe S6.Quantitative performance metrics of five radiomic models constructed using the clinical signature in the training and test cohorts.

| **model_name** | **Accuracy** | **AUC** | **95% CI** | **Sensitivity** | **Specificity** | **PPV** | **NPV** | **Cohort** |
| --- | --- | --- | --- | --- | --- | --- | --- | --- |
| LR | 0.683 | 0.726 | 0.665–0.788 | 0.681 | 0.684 | 0.241 | 0.936 | train |
| LR | 0.652 | 0.685 | 0.544– 0.825 | 0.625 | 0.654 | 0.119 | 0.959 | test |
| RandomForest | 0.730 | 0.747 | 0.691– 0.803 | 0.580 | 0.752 | 0.256 | 0.924 | train |
| RandomForest | 0.530 | 0.659 | 0.534– 0.783 | 0.812 | 0.509 | 0.110 | 0.973 | test |
| ExtraTrees | 0.631 | 0.631 | 0.562 –0.700 | 0.507 | 0.650 | 0.176 | 0.899 | train |
| ExtraTrees | 0.626 | 0.647 | 0.493–0.801 | 0.562 | 0.631 | 0.102 | 0.951 | test |
| XGBoost | 0.724 | 0.747 | 0.688–0.805 | 0.681 | 0.731 | 0.272 | 0.940 | train |
| XGBoost | 0.504 | 0.700 | 0.600–0.800 | 0.875 | 0.477 | 0.111 | 0.981 | test |
| LightGBM | 0.691 | 0.716 | 0.659 –0.773 | 0.710 | 0.688 | 0.251 | 0.942 | train |
| LightGBM | 0.500 | 0.693 | 0.587– 0.799 | 0.875 | 0.472 | 0.110 | 0.981 | test |

Figure S7.ROC curves of five different radiomics classifiers based on clinical signature for prediction in training and test datasets.

Table S7. Completed TRIPOD-AI reporting checklist. Corresponding locations of each checklist item in the revised manuscript are marked.

| **Section/Topic** | **Item** | **Checklist Item** | **Page** |
| --- | --- | --- | --- |
| **Title and abstract** | | | |
| Title | 1 | Identify the study as developing and/or validating a multivariable prediction model, the target population, and the outcome to be predicted. | 1 |
| Abstract | 2 | Provide a summary of objectives, study design, setting, participants, sample size, predictors, outcome, statistical analysis, results, and conclusions. | 2 |
| **Introduction** | | | |
| Background and objectives | 3a | Explain the medical context (including whether diagnostic or prognostic) and rationale for developing or validating the multivariable prediction model, including references to existing models. | 3 |
|  | 3b | Specify the objectives, including whether the study describes the development or validation of the model or both. | 3–4 |
| **Methods** | | | |
| Source of data | 4a | Describe the study design or source of data (e.g., randomized trial, cohort, or registry data), separately for the development and validation data sets, if applicable. | 4 |
|  | 4b | Specify the key study dates, including start of accrual; end of accrual; and, if applicable, end of follow-up. | 4 |
| Participants | 5a | Specify key elements of the study setting (e.g., primary care, secondary care, general population) including number and location of centres. | 4 |
|  | 5b | Describe eligibility criteria for participants. | 4 |
|  | 5c | Give details of treatments received, if relevant. | Not applicable (retrospective observational study; no targeted intervention was evaluated) |
| Outcome | 6a | Clearly define the outcome that is predicted by the prediction model, including how and when assessed. | 4 |
|  | 6b | Report any actions to blind assessment of the outcome to be predicted. | 4 (outcome was ascertained from standardized clinical records; ROI delineators were blinded to outcome status, see page 6) |
| Predictors | 7a | Clearly define all predictors used in developing or validating the multivariable prediction model, including how and when they were measured. | 5–7 |
|  | 7b | Report any actions to blind assessment of predictors for the outcome and other predictors. | 6 |
| Sample size | 8 | Explain how the study size was arrived at. | 4, 7 |
| Missing data | 9 | Describe how missing data were handled (e.g., complete-case analysis, single imputation, multiple imputation) with details of any imputation method. | 4 (complete-case analysis; cases with incomplete follow-up data were excluded) |
| Statistical analysis methods | 10a | Describe how predictors were handled in the analyses. | 7–8 |
|  | 10b | Specify type of model, all model-building procedures (including any predictor selection), and method for internal validation. | 8–9 |
|  | 10d | Specify all measures used to assess model performance and, if relevant, to compare multiple models. | 9 |
| Risk groups | 11 | Provide details on how risk groups were created, if done. | 9 (continuous risk estimated via nomogram; no predefined discrete risk subgroups) |
| **Results** | | | |
| Participants | 13a | Describe the flow of participants through the study, including the number of participants with and without the outcome and, if applicable, a summary of the follow-up time. A diagram may be helpful. | 10 |
|  | 13b | Describe the characteristics of the participants (basic demographics, clinical features, available predictors), including the number of participants with missing data for predictors and outcome. | 10–11 |
| Model development | 14a | Specify the number of participants and outcome events in each analysis. | 12–14 |
|  | 14b | If done, report the unadjusted association between each candidate predictor and outcome. | 11–12 |
| Model specification | 15a | Present the full prediction model to allow predictions for individuals (i.e., all regression coefficients, and model intercept or baseline survival at a given time point). | 12, 16 |
|  | 15b | Explain how to the use the prediction model. | 16 |
| Model performance | 16 | Report performance measures (with CIs) for the prediction model. | 13–15 |
| **Discussion** | | | |
| Limitations | 18 | Discuss any limitations of the study (such as nonrepresentative sample, few events per predictor, missing data). | 19–20 |
| Interpretation | 19b | Give an overall interpretation of the results, considering objectives, limitations, and results from similar studies, and other relevant evidence. | 17–19 |
| Implications | 20 | Discuss the potential clinical use of the model and implications for future research. | 20 |
| **Other information** | | | |
| Supplementary information | 21 | Provide information about the availability of supplementary resources, such as study protocol, Web calculator, and data sets. | 9 |
| Funding | 22 | Give the source of funding and the role of the funders for the present study. | 21 |

Table S8. Calibration performance metrics of six predictive models across training and test cohorts

| **Model** | **Cohort** | **Calibration Slope** | **Calibration Intercept** | **Hosmer-Lemeshow P-value** |
| --- | --- | --- | --- | --- |
| Clinical | Training | 1.412 | 0.315 | 0.482 |
| Clinical | Test | 1.568 | -0.463 | 0.295 |
| Intra-infarction Radiomics | Training | 1.351 | 0.264 | 0.551 |
| Intra-infarction Radiomics | Test | 1.477 | -0.401 | 0.346 |
| Peri1mm | Training | 1.436 | 0.328 | 0.467 |
| Peri1mm | Test | 1.541 | -0.449 | 0.308 |
| Peri2mm | Training | 1.39 | 0.297 | 0.513 |
| Peri2mm | Test | 1.514 | -0.432 | 0.321 |
| Peri3mm | Training | 1.455 | 0.341 | 0.443 |
| Peri3mm | Test | 1.592 | -0.486 | 0.274 |
| Combined | Training | 1.375 | 0.281 | 0.536 |
| Combined | Test | 1.502 | -0.418 | 0.329 |
